# Contrast-Enhanced Harmonic Endoscopic Ultrasound-Guided Fine-Needle Aspiration versus Standard Fine-Needle Aspiration in Pancreatic Masses: A Propensity Score Analysis

**DOI:** 10.3390/diagnostics10100792

**Published:** 2020-10-06

**Authors:** Antonio Facciorusso, Christian Cotsoglou, Andrea Chierici, Ruxandra Mare, Stefano Francesco Crinò, Nicola Muscatiello

**Affiliations:** 1 Gastroenterology Unit, Department of Medical Sciences, Ospedali Riuniti di Foggia, 71122 Foggia, Italy; antonio.facciorusso@virgilio.it; 2 General Surgery Department, ASST-Vimercate, 20871 Vimercate, Italy; christian.cotsoglou@asst-vimercate.it (C.C.); andreapiero.chierici@ast-vimercate.it (A.C.); 3 Department of Internal Medicine II, Gastroenterology Unit, “Victor Babes” University of Medicine and Pharmacy, 300226 Timisoara, Romania; ruxandra.mare@gmail.com; 4 Department of Medicine, Gastroenterology and Digestive Endoscopy Unit, The Pancreas Institute, University Hospital of Verona, 37100 Verona, Italy; stefanocrino@hotmail.com

**Keywords:** endoscopic ultrasound (EUS), fine-needle aspiration (FNA), pancreas, endoscopy, contrast-enhanced fine-needle aspiration (CH-FNA)

## Abstract

Background: Whether endoscopic ultrasound (EUS) contrast-enhanced fine-needle aspiration (CH-EUS-FNA) determines superior results in comparison to standard EUS-FNA in tissue acquisition of pancreatic masses remains unclear. The aim of this study was to compare these two techniques on a series of patients with solid pancreatic lesions. Methods: 362 patients underwent EUS-FNA (2008–2019), after the propensity score matching of two groups were compared; 103 treated with CH-EUS-FNA (group 1) and 103 with standard EUS-FNA (group 2). The primary outcome was the diagnostic accuracy. Secondary outcomes were sensitivity, specificity, and sample adequacy. Results: Diagnostic sensitivity was 87.6% in group 1 and 80% in group 2 (*p* = 0.18). The negative predictive value was 56% in group 1 and 41.5% in group 2 (*p* = 0.06). The specificity and positive predictive values were 100% for both groups. Diagnostic accuracy was 89.3% and 82.5%, respectively (*p* = 0.40). Sample adequacy was 94.1% in group 1 and 91.2% in group 2 (*p* = 0.42). The rate of adequate core histologic samples was 33% and 28.1%, respectively (*p* = 0.44), and the number of needle passes to obtain adequate samples were 2.4 ± 0.6 and 2.7 ± 0.8, respectively (*p* = 0.76). These findings were confirmed in subgroup analyses, conducted according to lesion size and contrast enhancement pattern. Conclusions: CH-EUS-FNA does not appear to be superior to standard EUS-FNA in patients with pancreatic masses.

## 1. Introduction

Endoscopic ultrasound-guided tissue acquisition (EUS-TA) for cytology by means of fine-needle aspiration (FNA) represents a pivotal technique in the assessment of pancreatic masses [1]. However, several factors have been described to influence the diagnostic performance of EUS-TA, thus determining a widely variable diagnostic sensitivity, ranging from 78–100% [2]. Among these variables potentially able to impact diagnostic characteristics of EUS-TA, use of suction, stylet [3], the fanning technique [4], rapid on-site cytopathology evaluation (ROSE) [5], needle caliper (19G vs. 22G vs. 25G) [6], and type of needle (FNA and fine needle biopsy (FNB)) [7,8] have been extensively studied but none of them were found to significantly influence the final outcomes so far.

While diagnostic specificity of EUS-FNA is usually excellent (around 100% in the published series), the most important pitfall associated with this procedure is a false-negative diagnosis that has the potential to delay patient care and negatively impact patient outcomes.

In order to increase EUS-FNA sensitivity and accuracy for the evaluation of pancreatic masses, specific techniques already used in other fields (for instance in liver oncology) have been developed, such as real-time elastography EUS (RTE-EUS)-FNA [9] and contrast-enhanced harmonic EUS (CH-EUS)-FNA [10,11].

Due to their interesting performance in terms of negative predictive value (NPV), these two techniques (either isolated or combined) raised hopes to ameliorate the diagnostic algorithm of pancreatic masses, thus increasing the quality of EUS procedures [12].

In particular, CH-EUS, with a second-generation ultrasound contrast agent, can detect signals from microbubbles in vessels with a very slow flow without Doppler-related artifacts and can be used to characterize microvascularity in the pancreas, thus aiding in the differentiation of solid pancreatic masses [13].

As most adenocarcinomas are hypoenhanced, CH-EUS can help to identify the target for EUS-FNA, with easier avoidance of anechoic areas and vessels inside the tumor [14]. Several reports showed that targeting FNA through the hypoenhanced area of pancreatic masses can lead to favorable diagnostic performances, with the greater magnitude of benefit in isoechoic lesions, which are undetectable with standard EUS but only suspected due to the “ductal cutoff sign” [15].

Five series directly compared CH-EUS-FNA to standard EUS-FNA [10,11,16,17,18], of which two randomized-controlled trials (RCTs) [10,16] have been published so far with conflicting results. Therefore, whether CH-EUS-FNA might lead to a significant increase in diagnostic sensitivity and accuracy as compared to EUS-FNA remains an open debate.

The aim of this study was to examine the outcomes of patients undergoing CH-EUS-FNA, as compared to an historical series of patients treated with standard EUS-FNA at our center, and to compare the efficacy and safety of these two different EUS-TA techniques.

## 2. Materials and Methods

### 2.1. Patients

From a prospectively collected database of 426 patients with suspected pancreatic masses by cross-sectional imaging examination, referred to our center between January 2008 and December 2019 to be evaluated by means of EUS, data regarding 362 consecutive patients who underwent EUS-FNA of pancreatic masses were reviewed. Institutional Review Board approbation for this retrospective report was obtained.

The following exclusion criteria were used: (1) reluctance to receive EUS-FNA or inability to sign informed consent; (2) high procedural risks; (3) clear indication to surgical treatment; and (4) coagulopathy (international normalized ratio > 1.5, platelets < 50,000). Patients in antithrombotic treatment suspended the anticoagulant/antiaggregant agent and underwent bridging therapy, with enoxaparin when necessary. No intravenous prophylactic antibiotics before EUS-FNA were administered, as per current guidelines [19].

All procedures were performed by a board-certified gastroenterologist with 20 years of experience (N.M.).

After exclusion of subjects who did not fulfill the inclusion criteria, the study population included two groups of patients: 250 who underwent standard EUS-FNA and 112 were treated with CH-EUS-FNA.

### 2.2. EUS Technique

The technique used for diagnostic and interventional EUS at our center has been described elsewhere [20,21,22,23,24].

Briefly, under sedation with propofol (Diprivan^®^, AstraZeneca, London, UK), EUS was conducted with a Pentax FG-36UA ultrasound endoscope (Pentax Europe, Ltd., Hamburg, Germany) using a curved-array transducer. Once in the stomach, the endoscopic ultrasound probe was located in contact with the gastric wall and then slowly advanced up to duodenum when necessary. A 22 G needle (EchoTip Ultra^®^, Cook Medical Inc, Bloomington, Indiana, USA), with a central stylet to protect the aspiration channel of the needle, was introduced though the endoscope’s working channel. A trans-duodenal approach was used for lesions of the pancreatic head and uncinate process, whereas the trans-gastric approach was used for lesions of the body or tail. The needle was inserted into the lesion and, immediately after the procedure, the stylet was removed. More than 10 to-and-fro movements were made within the lesion, and aspiration was obtained with a 10 cm^3^ suction syringe applied to the hub of the FNA device.

**Figure 1:**
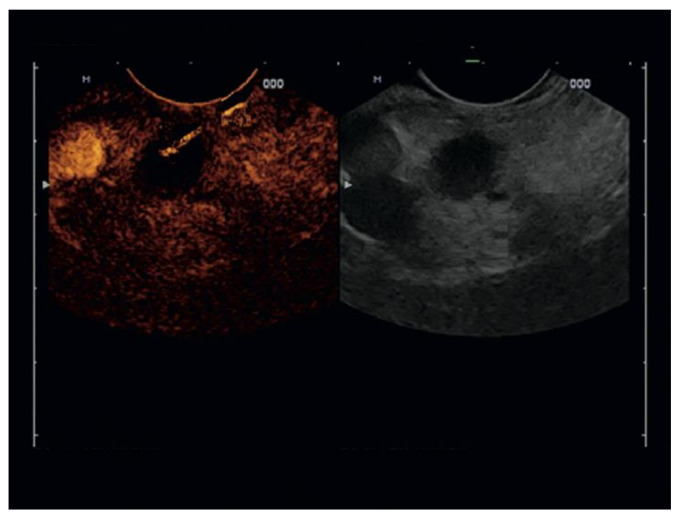
Endoscopic ultrasound contrast-enhanced fine-needle aspiration of a pancreatic adenocarcinoma. The hypoenhancement area of the lesion was selected as a target area for fine-needle aspiration.

In the CH-EUS-FNA group, lesions were imaged in real-time with the extended pure harmonic detection and simultaneously monitored by the fundamental B mode. An intravenous injection of 4.8 mL of SonoVue^®^ (Bracco, Milan, Italy) was administered through an antecubital vein with a 20-G catheter, followed by a 20-mL saline flush. Pancreatic lesion enhancement was compared with the adjacent pancreatic parenchyma, and three patterns were differentiated as hypo-, iso-, or hyperenhancement. According to the current guidelines, two phases were described: a) the arterial phase, starting from around 10–20 s until around 35–40 s after contrast injection, and b) the venous phase, starting from around 30–45 s after contrast injection [25]. When a pancreatic lesion was diagnosed as pancreatic adenocarcinoma by CH-EUS, the hypoenhancement area of the lesion was selected as a target area for FNA (Figure 1); on the other hand, when the CH-EUS diagnosis was focal pancreatitis or a neuroendocrine tumor, the iso- or hyperenhancement area of the lesion was selected, according to the current literature [10,11,16,17,18]. The FNA site was monitored simultaneously by endosonography.

At the end of the procedure, the needle was retracted. After being grossly checked for adequacy, samples were prepared for cytological examination with Papanicolaou staining, and eventual further passes were performed if samples were not considered adequate. An on-site cytologist was not available.

The cytologic diagnosis and sample adequacy were reviewed by two cytopathologists blinded to the EUS-TA technique adopted.

Patients were continuously monitored during the procedure by a board-certified anesthesiologist with an automated noninvasive blood pressure device, electrocardiogram tracing, and pulse oximetry. Depending on the complexity of the procedure and comorbidity, patients were either hospitalized for observation for 24 h or had the procedure in the day hospital. In both cases, the monitoring protocol was the same.

### 2.3. Outcomes

Data were collected by a resident (A.C.), wh was blinded to the technique used.

The final diagnosis of pancreatic malignancy was based either on surgical pathology for patients who underwent surgery or on the clinical course (clinical progression or death from malignancy) during a follow-up of 12 months. Absence of disease progression or resolution of the imaging (mainly a CT-scan, RMI, or EUS) or clinical changes were indicative of the diagnosis of benign disease.

The primary outcome was diagnostic accuracy, defined as the summary of true positives (TPs) and true negatives (TNs) on the total number of patients. The secondary outcomes were diagnostic sensitivity, computed as the proportion of positives correctly identified by the test (TPs) on the prevalence of disease in the study cohort ((TPs and false negatives (FNs)), diagnostic specificity, calculated as the proportion of negatives correctly identified as such (TNs) among the patients who were not affected by the disease in the study cohort ((TNs and false positives (FPs)), and sample adequacy, defined as the proportion of samples defined as adequate for cytological diagnosis. Additional outcomes were histologic core procurement, defined as the proportion of patients with samples adequate for histological diagnosis, number of needle passes needed to obtain adequate samples, positive likelihood ratio, defined as sensitivity/false positive rate, and negative likelihood ratio, defined as false negative rate/specificity.

Adverse events (AEs) were evaluated during the procedure and before discharge. A severe adverse event was defined as one that required hospitalization, was life-threatening, or resulted in death or disability.

### 2.4. Statistical Analysis

Categorical variables were reported as the number of cases and percentage, and differences between groups were compared using the Chi-square and McNemar analysis before and after matching, respectively.

Continuous variables were expressed as mean and standard deviation, and differences between groups were explored by the Mann–Whitney and Wilkoxon rank tests before and after matching, respectively. All analyses were 2-tailed, and the threshold of significance was assessed at ≤0.05.

To overcome biases owing to the different distribution of covariates, a 1-to-1 match was created using propensity score analysis. The propensity score represents the probability of each individual patient being assigned to a particular condition in a study given a set of known covariates [26].

The propensity score model was built upon a multivariate logistic regression analysis to predict the probability of each individual patient being submitted to one of the two EUS-TA techniques, based on several demographic and clinical covariates, namely, age, gender, lesion size, location, and final diagnosis.

The predictive values were then used to obtain a 1-to-1 match by using the nearest neighbor matching within a specified caliper distance. Nearest neighbor matching within a specified caliper distance selects an untreated subject for matching whose propensity score is closest to that of the treated subject (“nearest neighbor matching” approach), and the further restriction being the absolute difference in the propensity scores of matched subjects, must be below a pre-specified threshold (the caliper distance) [26,27]. Thus, the propensity score could not be matched for some patients because a greater caliper distance was excluded from further analysis. As suggested by Austin, a caliper of width equal to 0.2 of the standard deviation of the logit of the propensity score was used, as this value has been found to minimize the mean squared error of the estimated treatment effect [27,28].

Subgroup analysis was based on mass size (≤1.5 cm versus >1.5 cm and ≤2 cm versus >2 cm) and, within the CH-EUS-FNA group, based on the contrast enhancement pattern (hypoenhanced versus iso/hyperenhanced lesions).

The statistical analysis was performed using the MatchIt package in R Statistical Software 3.0.2 (Foundation for Statistical Computing, Vienna, Austria).

## 3. Results

### 3.1. Patients

The baseline characteristics of the whole series of 362 patients who underwent EUS-guided tissue acquisition of pancreatic masses at our center are reported in Table 1.

Before propensity score matching, Group 1, namely, patients treated with CH-EUS-FNA, included 112 patients, whereas 250 subjects underwent traditional EUS-FNA (Group 2). As reported in Table 1, the mean age was 67 years in Group 1 and 65 years in Group 2 (*p* = 0.58), whereas the number of male patients were 65 (68%) and 150 (60%) in the two groups, respectively (*p* = 0.72). Most of the sampled lesions were located in the head/uncinate process (69.6% in Group 1 and 66.8% in Group 2, *p* = 0.59), with a mean size of 3.4 and 3.1 cm in the two groups, respectively (*p* = 0.12). Final diagnosis was mainly adenocarcinoma (72.3% in Group 1 and 74% in Group 2, *p* = 0.62).

**Figure 2:**
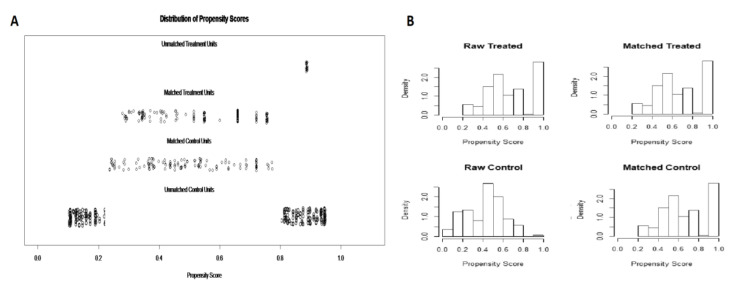
Propensity score matching. Out of the initial 362 patients, after 1-to-1 propensity score caliper matching, 206 patients were selected for comparison: 103 patients who were treated with endoscopic ultrasound contrast-enhanced fine-needle aspiration and 103 patients who were treated with endoscopic ultrasound standard fine-needle aspiration. (**A**) Propensity score matching jitter plot; (**B**) propensity score matching histogram.

After 1-to-1 propensity score matching, 206 patients were selected for comparison: 103 subjects who were treated with CH-EUS-FNA (Group 1) and 103 patients who underwent standard EUS-FNA (Group 2). Details of propensity score matching are shown in Figure 2A,B.

The characteristics of these propensity score-matched patients are reported in Table 1.

The mean age was 66 years in the two groups (*p* = 0.91), and more than half of the treated patients were male (55.3% in Group 1 and 54.3% in Group 2; *p* = 0.88). The vast majority of sampled lesions were located in the head/uncinate process (68.9% in both groups, *p* = 1.0), with a mean size of 3.2 cm (*p* = 0.93). Again, adenocarcinoma was the most frequent diagnosis (73.9% in both groups, *p* = 1.0).

### 3.2. Diagnostic Performance

A detailed list of the study outcomes is reported in Table 2.

A total of 78 patients in Group 1 and 72 patients in Group 2 were correctly diagnosed as true positive (i.e., diagnosis confirmed in surgical histology or clinical follow-up), whereas 14 and 13 patients were correctly diagnosed as true negative (i.e., absence of disease confirmed in surgical histology or clinical follow-up). On the other hand, 11 patients in group 1 and 18 patients in Group 2 were categorized as false negative (of which there were 6 and 9 inadequate samples in the two groups, respectively, *p* = 0.16). No false positives were observed in either of the two study groups.

As a consequence, diagnostic sensitivity was 87.6% (95% confidence interval 78.9–93.6%) in Group 1 and 80% (70.2–87.7%) in Group 2 (*p* = 0.18), whereas specificity was 100% in both groups.

The negative predictive value was 56% (42.2–68.8%) in Group 1 and 41.5% (32.3–52.2%) in Group 2 (*p* = 0.06), the positive predictive value was 100% in both groups, and the diagnostic accuracy was 89.3% (81.6–94.5%) and 82.5% (73.8–89.3%) in the two groups, respectively (*p* = 0.11).

The sample adequacy was 94.1% in Group 1 and 91.2% in Group 2 (*p* = 0.42), whereas the rate of adequate core histologic samples was 33% and 28.1% in the two groups, respectively (*p* = 0.44).

Number of needle passes needed to obtain adequate samples was similar in the two groups (2.4 ± 0.6 and 2.7 ± 0.8, respectively; *p* = 0.76).

No serious adverse event was registered in the study.

### 3.3. Subgroup Analysis

Subgroup data are detailed in Table 3.

In the subgroup of patients with greater lesions (>1.5 cm: 69 patients in both groups), diagnostic sensitivity was 88% in Group 1 and 80.1% in Group 2 (*p* = 0.21), accuracy was 90.3% and 83.1% in the two groups, respectively (*p* = 0.35), and sample adequacy was 96% in Group 1 and 93% in Group 2 (*p* = 0.44).

Similar results were observed in the subgroups, defined according to a different size cut-off (≤2 cm (45 patients in both groups) versus >2 cm (58 patients in both groups): Table 3).

Within the group of patients who underwent CH-EUS-FNA, 73 subjects presented hypoenhanced lesions and 30 subjects presented iso/hyperenhanced masses. In the subgroup of hypoenhanced lesions, diagnostic sensitivity was 86.5%, accuracy was 88.7%, and adequacy was 93.8%. In the subgroup of iso/hyperenhanced lesions, sensitivity, accuracy, and adequacy were 88.9%, 89.9%, and 94.7%, respectively. No differences between the two subgroups in any of the aforementioned performance measures were registered (Table 3).

## 4. Discussion

In spite of the recent advancements in the field of EUS-guided tissue acquisition [29] of pancreatic masses, the relatively high rate of false negatives still represents an unsolved issue. The presence of fibrosis and necrosis inside the pancreatic mass is responsible for most of these false negative cases and requires frequently multiple FNA passes to achieve adequate samples.

Contrast enhancement enables a better identification of the targeted area for FNA in pancreatic lesions and might decrease the false negative results by avoiding the areas rich in blood, necrosis, and fibrosis; in fact, necrosis, vessels, and cystic non-enhanced areas can be more easily avoided and the lesion could be better defined with CH-EUS-FNA, so the best area for sampling can be more precisely targeted and with more visibility [30]. Pancreatic adenocarcinoma usually appears as an hypoenhanced lesion, where the needle should target the least enhanced part of the mass, thus avoiding both the more enhanced area, likely rich in vessels, and the non-enhanced area, which is suggestive of necrosis [30]. On the other hand, benign lesions and neuroendocrine tumors (NETs) are usually iso/hyperenhanced lesions, where the non-enhanced area should be avoided as it could represent an area of hemorrhage or necrosis [30].

Although this method is simple and was not found to significantly prolong the duration of the procedure, there is only limited and conflicting evidence on the comparative efficacy of CH-EUS-FNA as compared to standard EUS-FNA in patients with pancreatic masses. Therefore, we decided to examine the performance of CH-EUS-FNA in our series of patients with pancreatic masses and compare it to an historical cohort of patients treated with standard EUS-guided fine-needle aspiration. In order to overcome the potential biases related to the retrospective nature of the study and properly take into account all confounding variables, we performed a propensity score matching analysis on the basis of several demographic and procedural covariates, thus, two perfectly balanced treatment groups were obtained (Table 1).

Diagnostic performance measures were absolutely comparable between the two groups. In particular, diagnostic sensitivity was 87.6% in Group 1 and 80% in Group 2 (*p* = 0.18) and diagnostic accuracy was 89.3% and 82.5% in the two groups, respectively (*p* = 0.11). These results are in line with previous reports [10,17] and confirm that CH-EUS-FNA is not able to significantly increase the performance of EUS-FNA in patients with solid pancreatic lesions. As expected, specificity and positive predictive values were 100% in both groups.

Similarly, CH-EUS-FNA did not significantly increase the rate of adequate samples, neither when considering cytology (94.1% in Group 1 and 91.2% in Group 2, *p* = 0.42), nor when considering core histologic samples (33% and 28.1% in the two groups, *p* = 0.44).

Targeting FNA based on the contrast enhancement pattern could reasonably be considered able to decrease the number of needle passes needed to obtain adequate samples; instead, no difference concerning this parameter was observed in our study (2.4 ± 0.6 and 2.7 ± 0.8, respectively; *p* = 0.76), again in line with the current literature [10,17,18].

Moreover, there were no significant differences in diagnostic ability between the two methods in patients with small lesions (≤1.5 cm and ≤2 cm). This suggests that CH-EUS-FNA is unnecessary for patients with small lesions before performing EUS-FNA. Even the contrast enhancement pattern was irrelevant in determining higher accuracy and sensitivity with CH-EUS-FNA. Again, this finding confirms previous reports [10]. No serious adverse event was registered in the study, thus confirming the safety of EUS-FNA in patients with pancreatic masses.

Our results confirm those of a large cross-over RCT [10] and point out the fact that CH-EUS-FNA cannot fill the gap between the diagnostic performance obtained with EUS-FNA and newer EUS-guided FNB needles [8].

This study has a number of strengths: First, it is one of the largest published series directly comparing the efficacy of the two techniques and the parallel design allows a better comparison between the two study groups. Secondly, the accurate statistical design and the completeness of the collected data strengthen the results of our analysis. Thirdly, the unicentricity of the current study is a guarantee against eventual biases due to different treatment procedures or endoscopic trainings.

Nevertheless, the paper has some weaknesses. Its main limitation is the retrospective nature of the study, which could have led to selection biases. However, a propensity score matching analysis based on the baseline covariates known to influence diagnostic outcomes was performed in order to obviate to the aforementioned bias. In addition, several subgroups analyses were performed to identify eventual sources of heterogeneity. Second, only a single needle size (22G FNA) was tested; therefore, our results should not be considered applicable to other devices, such as 25G and 19G, although previous series did not seem to suggest significant differences based on needle size [16]. Finally, cost-effectiveness analysis was beyond the scope of the current study.

## 5. Conclusions

Despite such limitations, our analysis seems to suggest that CH-EUS-FNA and standard EUS-FNA have similar diagnostic accuracy in patients with pancreatic masses. Although it is unlikely that contrast enhancement or elastography-guided FNA could fill the performance gap with newer FNB needles, further research, in particular based on large multicentric RCTs, is needed to confirm our results.

## Figures and Tables

**Table 1:** Baseline characteristics of the patients recruited in the study.

Baseline Patients’ Characteristics before Propensity Score Matching			
Variable	CH-EUS-FNA (*n* = 112)	EUS-FNA (*n* = 250)	*p* Value
Age (years)	67 ± 5	65 ± 10	0.58
Gender: Male	65 (58%)	150 (60%)	0.72
Mass location Head/uncinate Body/tail	78 (69.6%) 34 (30.4%)	167 (66.8%) 83 (33.2%)	0.59
Mass size (cm)	3.4 ± 1.1	3.1 ± 0.9	0.12
Diagnosis adenocarcinoma NET benign	81 (72.3%) 9 (8%) 22 (19.7%)	185 (74%) 25 (10%) 40 (16%)	0.62
**Baseline Patients’ Characteristics after Propensity Score Matching**			
**Variable**	**CH-EUS-FNA (*n* = 103)**	**EUS-FNA (*n* = 103)**	***p* Value**
Age (years)	66 ± 6	66 ± 8	0.91
Gender: Male	57 (55.3%)	56 (54.3%)	0.88
Mass location Head/uncinate Body/tail	71 (68.9%) 32 (31.1%)	71 (68.9%) 32 (31.1%)	1.0
Mass size (cm)	3.2 ± 1.1	3.2 ± 1	0.93
Diagnosis adenocarcinoma NET benign	76 (73.9%) 7 (6.7%) 20 (19.4%)	76 (73.9%) 7 (6.7%) 20 (19.4%)	1.0

Continuous variables were reported as mean values and standard deviations. The following variables were selected for propensity score calculation: age, gender, lesion size, location, and final diagnosis. Abbreviations: Contrast-enhanced (CH); endoscopic ultrasound (EUS); fine-needle aspiration (FNA); neuroendocrine tumor (NET).

**Table 2:** Diagnostic performance of the two techniques.

	CH-EUS-FNA (103 Patients)	EUS-FNA (103 Patients)	*p*-Value ^a^
True positive	78	72	0.34
True negative	14	13	0.83
False positive	0	0	1.0
False negative/inadequate samples	11	18	0.16
Sensitivity	87.6% (78.9–93.6%)	80% (70.2–87.7%)	0.18
Specificity	100%	100%	1.0
PPV	100%	100%	1.0
NPV	56% (42.2–68.8%)	41.5% (32.3–52.2%)	0.06
Accuracy	89.3% (81.6–94.5%)	82.5% (73.8–89.3%)	0.11
Sample adequacy	94.1%	91.2%	0.42
Histologic core procurement	33%	28.1%	0.44
Number of passes	2.4 ± 0.6	2.7 ± 0.8	0.76

Variables are expressed as the absolute number (percentage). ^a^ Calculated by means of the McNemar test. Abbreviations: contrast-enhanced (CH); endoscopic ultrasound (EUS); fine-needle aspiration (FNA); negative predictive value (NPV); positive predictive value (PPV).

**Table 3:** Subgroup analysis.

	CH-EUS-FNA	EUS-FNA	*p*-Value ^a^
Sensitivity			
≤1.5 cm (34 patients)	87%	80.4%	0.20
>1.5 cm (69 patients)	88%	80.1%	0.21
≤2 cm (45 patients)	87.3%	81.2%	0.28
>2 cm (58 patients)	88.1%	80.5%	0.37
Hypoenhanced (73 patients)	86.5%	--	0.74 *
Iso/Hyperenhanced (30 patients)	88.9%	--	
Accuracy			
≤1.5 cm (34 patients)	87.5%	82%	0.39
>1.5 cm (69 patients)	90.3%	83.1%	0.35
≤2 cm (45 patients)	88.5%	81.3%	0.15
>2 cm (58 patients)	90.1%	83.8%	0.27
Hypoenhanced (73 patients)	88.7%	--	0.86 *
Iso/Hyperenhanced (30 patients)	89.9%	--	
Sample adequacy			
≤1.5 cm (34 patients)	92.4%	89%	0.63
>1.5 cm (69 patients)	96%	93%	0.44
≤2 cm (45 patients)	93.4%	90.3%	0.59
>2 cm (58 patients)	94.7%	92.4%	0.61
Hypoenhanced (73 patients)	93.8%	--	0.86 *
Iso/Hyperenhanced (30 patients)	94.7%	--	

Variables are expressed as the absolute number (percentage). ^a^ Calculated by means of the McNemar test. * Refers to the comparison between hypoenhanced and iso/hyperenhanced lesions within the CH-EUS-FNA group. Abbreviations: contrast-enhanced (CH); endoscopic ultrasound (EUS); fine-needle aspiration (FNA).In the subgroup of patients with small lesions (≤1.5 cm: 34 patients in Group 1 and 34 patients in Group 2), diagnostic sensitivity was 87% in Group 1 and 80.4% in Group 2 (*p* = 0.20), accuracy was 87.5% and 82% in the two groups, respectively (*p* = 0.39), and sample adequacy was 92.4% in Group 1 and 89% in Group 2 (*p* = 0.63).
